# Phytohormone and Putative Defense Gene Expression Differentiates the Response of ‘Hayward’ Kiwifruit to Psa and Pfm Infections

**DOI:** 10.3389/fpls.2017.01366

**Published:** 2017-08-04

**Authors:** Kirstin V. Wurms, Allan J. Hardaker, Annette Ah Chee, Judith Bowen, Janet Phipps, Joseph Taylor, Dwayne Jensen, Janine Cooney, Mark Wohlers, Tony Reglinski

**Affiliations:** ^1^The New Zealand Institute for Plant & Food Research Limited Hamilton, New Zealand; ^2^The New Zealand Institute for Plant & Food Research Limited Auckland, New Zealand

**Keywords:** bacterial canker, defense gene expression, host resistance, phytohormone regulation, plant–pathogen interactions

## Abstract

*Pseudomonas syringae* pv. *actinidiae* (Psa) and *Pseudomonas syringae* pv. *actinidifoliorum* (Pfm) are closely related pathovars infecting kiwifruit, but Psa is considered one of the most important global pathogens, whereas Pfm is not. In this study of *Actinidia deliciosa* ‘Hayward’ responses to the two pathovars, the objective was to test whether differences in plant defense responses mounted against the two pathovars correlated with the contrasting severity of the symptoms caused by them. Results showed that Psa infections were always more severe than Pfm infections, and were associated with highly localized, differential expression of phytohormones and putative defense gene transcripts in stem tissue closest to the inoculation site. Phytohormone concentrations of jasmonic acid (JA), jasmonate isoleucine (JA-Ile), salicylic acid (SA) and abscisic acid were always greater in stem tissue than in leaves, and leaf phytohormones were not affected by pathogen inoculation. Pfm inoculation induced a threefold increase in SA in stems relative to Psa inoculation, and a smaller 1.6-fold induction of JA. Transcript expression showed no effect of inoculation in leaves, but Pfm inoculation resulted in the greatest elevation of the SA marker genes, PR1 and glucan endo-1,3-beta-glucosidase (β-1,3-glucosidase) (32- and 25-fold increases, respectively) in stem tissue surrounding the inoculation site. Pfm inoculation also produced a stronger response than Psa inoculation in localized stem tissue for the SA marker gene PR6, jasmonoyl-isoleucine-12-hydrolase (JIH1), which acts as a negative marker of the JA pathway, and APETALA2/Ethylene response factor 2 transcription factor (AP2 ERF2), which is involved in JA/SA crosstalk. WRKY40 transcription factor (a SA marker) was induced equally in stems by wounding (mock inoculation) and pathovar inoculation. Taken together, these results suggest that the host appears to mount a stronger, localized, SA-based defense response to Pfm than Psa.

## Introduction

*Pseudomonas syringae* pv. *actinidiae* (Psa), a gram negative bacterium responsible for bacterial canker, is considered to be one of the most devastating global pathogens of kiwifruit (*Actinidia deliciosa* and *A. chinensis*), (Vanneste, 2012; Michelotti et al., 2015). Psa biovar 3 is the biovar responsible for causing the recent worldwide outbreak of bacterial canker (Vanneste et al., 2013). *Pseudomonas syringae* pv. *actinidifoliorum* (Pfm) was originally thought to be a less virulent form of Psa that caused smaller lesions and localized infection compared with Psa, and was formerly classified as biovar 4, but it has been subsequently classified as a separate pathovar on the basis of differential biochemical, genetic and pathogenicity characteristics (Cunty et al., 2015).

Psa infected vines exhibit leaf spots, cane wilting and dieback, and cankers that are sometimes associated with exudate, and it spreads rapidly in suitable weather conditions, such as high rainfall and cooler temperatures in spring and autumn (Vanneste, 2013; Vanneste et al., 2013). Like most bacterial pathogens, effective control options for Psa are limited, and the mainstays of chemical control are copper pesticides and antibiotics (Reglinski et al., 2013). Disadvantages of using these products include phytotoxicity, heavy metal accumulation in the soil, and development of resistance.

Longer term, more sustainable control measures include breeding for resistance and the use of elicitors in integrated control programs to temporarily induce host defenses. Successful implementation of both resistance breeding and use of elicitors requires a thorough understanding of the mechanisms and genes involved. Breeding for resistance has to be carefully controlled so that other desirable characteristics such as high dry matter, for example, are not lost. Elicitors can induce different phytohormonal defense-signaling pathways (Romanazzi et al., 2016), such as the abscisic acid (ABA) pathway that is involved in generalized stress responses to abiotic and biotic factors (Ton et al., 2009), the salicylic acid (SA) pathway, which tends to be induced by biotrophic pathogens and sap-sucking insects (Loake and Grant, 2007; Erb et al., 2012), and the jasmonic acid (JA) pathway which responds to wounding, chewing insects and invasion by necrotrophic pathogens (Dar et al., 2015). There is often complex cross talk and sometimes antagonism between these pathways, for example, elevation of the SA pathway leading to suppression of the JA pathway and vice versa (Pieterse et al., 2012; Verma et al., 2016). Successful use of an elicitor requires identification of the pathways that are effective in the particular host–pathogen interaction being studied. An understanding of the mode of action of elicitors is equally important because efficacy under field conditions varies with cultivar, timing and frequency of application, and crop phenology (Walters et al., 2011; Bruce, 2014; Reglinski et al., 2014a). In addition, knowledge of the mechanisms involved is needed to minimize the metabolic costs associated with elicitor use, such as reduced fruit production or quality (Walters and Heil, 2007; Cipollini and Heil, 2010). Whilst there are published studies on elicitor-induced responses of different kiwifruit cultivars to Psa, which suggest predominance of the SA-mediated pathway (Petriccione et al., 2013, 2014; Cellini et al., 2014; Reglinski et al., 2014b), there has been no investigation of the molecular and biochemical basis of the variable host response to Psa and the closely related Pfm. Consequently the current study was carried out to address this gap in our knowledge. The study was performed on *Actinidia deliciosa* ‘Hayward’ because this is the most widely grown cultivar in the world (Nardozza et al., 2013). Specific objectives of this research were to elucidate phytohormone-mediated signaling pathways that play a key role in orchestrating the kiwifruit defense response to Psa and Pfm, and, if possible, to assist identification of molecular markers of plant defense for marker assisted selection (MAS) in breeding programs.

## Materials and Methods

### Plant Material

All the tissue cultured ‘Hayward’ kiwifruit plants used in these experiments came from Multiflora Laboratories Ltd. (Auckland, New Zealand). After arrival as rooted plantlets in agar growth medium in plastic tubs, each plantlet was individually exflasked into 30 mm Rockwool^®^ cubes (Rockwool BV, Netherlands) and stored in closed plastic containers for up to 2 weeks with gradual acclimatization to ambient temperature and humidity. They were then transferred to a containment glasshouse and maintained at 15–24°C, with a day length of approximately 14 h. A flood and drain system was used once daily to water the plantlets with a hydroponic nutrient solution, pH 6.2, containing 145 ppm nitrogen (plus extra nitrogen from the nitric acid used for pH correction), 32 ppm phosphorous, 190 ppm potassium, 103 ppm calcium, 28 ppm magnesium, 49 ppm sulfur, 3 ppm iron and trace elements (0.07–1.2 ppm) (PGO Horticulture Ltd., New Zealand). Plantlets were actively growing with 3–4 fully expanded leaves and were approximately 30 cm tall were when they were used for experiments.

### Inoculum Preparation and Inoculation Technique

Psa biovar 3 (strain #10627, described by Vanneste et al., 2013) and Pfm (ICMP strain 19098 obtained from the International Collection of Microorganisms from Plants held by Landcare Research, New Zealand) were plated onto King’s B medium supplemented with cycloheximide (0.018%) and boric acid (0.136%) and incubated at 25°C for 24 h. Bacterial colonies were resuspended in sterile water to a concentration of 10^9^ colony forming units (cfu)/ml. Concentration was estimated by measurements of optical density with subsequent measurement by plating out and performing colony counts.

Plants were inoculated by dipping a toothpick into the appropriate Psa or Pfm inoculum at 1 × 10^9^ cfu/ml of water, and pricking the stem of each plantlet at a single point 1 cm below the leaf petiole of the third unfurled leaf. For mock inoculations the toothpick was dipped in sterile water instead of inoculum.

### Disease Assessments

Disease severity was assessed by measuring the length (mm) of the lesion emanating from the inoculation point on each plant at several time points 13–29 days post inoculation (dpi). Lesion lengths do not necessarily correspond with bacterial titre, but the results of lesion length bioassays correlate positively with actual differences in Psa resistance observed in the field, and are used by the New Zealand kiwifruit industry to screen for Psa resistance in their breeding programs (Datson et al., 2015; Hoyte et al., 2015; Nardozza et al., 2015).

### Experiments

#### Experiment (Exp) 1 – Leaf Analysis

In this experiment, the response to stem infection by Psa versus Pfm was compared in ‘Hayward’ tissue cultured plants grown in Rockwool^®^ cubes.

The third unfurled leaf, situated 1 cm above the inoculation point on each plant was sampled at time zero (before inoculation) for untreated plants (to form a baseline for qPCR normalization), and 24 and 48 h after inoculation with water, Psa or Pfm for the remaining treatments (**Table 1**). Sampling times of 24 and 48 h post inoculation were shown to be the most suitable in a preliminary, unpublished time course experiment (data not shown). The sampled leaves from three plants per replicate were pooled, with the same tissue being used for biochemical and molecular analyses, and there were three biological replicates per treatment. Sampled leaves had their mid-vein removed then were snap frozen in liquid nitrogen and stored at -80°C until extraction for molecular assessments and for enzyme/hormone work. Actual concentrations of Psa and Pfm on the day of inoculation were 1.7 × 10^9^ cfu/ml and 1.0 × 10^9^ cfu/ml, respectively (the small difference between inoculum concentrations was not considered significant, given the heavy inoculum dose).

**Table 1:** Experiment 1 treatments applied to ‘Hayward’ tissue cultured plantlets in the glasshouse.

Treatment abbreviation	Treatment details	Designated use	Number of plants/rep	Number of biological reps
Baseline	Nil – untreated	Sample	3	3
Mock inoc 24	Water inoculated, sampled 24 hours post inoculation (hpi)	Sample	3	3
Psa 24	Psa inoculated, sampled 24 hpi	Sample	3	3
Pfm 24	Pfm inoculated, sampled 24 hpi	Sample	3	3
Mock inoc 48	Water inoculated, sampled 48 hpi	Sample	3	3
Psa 48	Psa inoculated, sampled 48 hpi	Sample	3	3
Pfm 48	Pfm inoculated, sampled 48 hpi	Sample	3	3
Mock inoc	Water inoculated	Disease	1	24
Psa	Psa inoculated	Disease	1	24
Pfm	Pfm inoculated	Disease	1	24

*Separate groups of plants were used for disease assessments (designated use = disease) and tissue sampling for biochemical and molecular analyses (designated use = sample). Disease assessments were made on whole plants (*n* = 24), and tissue sampling for biochemical and molecular analyses comprised pooling the third leaf of each of three plants per replicate (rep) and using the same tissue for both biochemical and molecular analyses (*n* = 3).*

Disease assessments were made, on separate plants to those used for biochemical and molecular analyses, 13, 21 and 29 dpi (**Table 1**).

#### Exp 2 – Leaf and Stem Analysis

A second experiment was designed as a follow up to the first trial to compare the ‘Hayward’ response to Psa versus Pfm in different tissue types. The treatments were similar to experiment one (**Table 2**), except that tissue sampling was carried out 48 hours post inoculation (hpi) at three different sites on each plantlet – these sites were designated “stem local”, “stem systemic” and “leaf”.

**Table 2:** Experiment 2 treatments applied to ‘Hayward’ tissue cultured plantlets in the glasshouse.

Treatment abbreviation	Treatment details	Designated use	Number of plants/ replicate (rep)	Number of biological reps
Baseline	Nil – untreated	Sample	5	3
Mock inoc 48	Water inoculated sampled 48 hours post inoculation (hpi)	Sample	5	3
Psa 48	Psa inoculated, sampled 48 hpi	Sample	5	3
Pfm 48	Pfm inoculated, sampled 48 hpi	Sample	5	3
Mock inoc	Water inoculated	Disease	1	10
Psa	Psa inoculated	Disease	1	10
Pfm	Pfm inoculated	Disease	1	10

*Separate groups of plants were used for disease assessments (*n* = 10) and tissue sampling for biochemical and molecular analyses (three tissue pools of five plants each, i.e., *n* = 3).*

For sampling of the stem tissue in the untreated plants, a 2 cm section of stem from the area designated for inoculation in all other treatments was removed for molecular analysis and a second 2 cm section immediately above this for biochemical measurements. For all other treatments, 3 cm × 1.5 cm stem sections per plant were removed. These three sections comprised a 1.5 cm length of stem tissue containing the inoculation site at its center (“stem local”) that was used for molecular analysis, the next 1.5 cm section of stem immediately above the “stem local” section that was used for biochemical analysis, and another 1.5 cm section immediately above the stem tissue sampled for biochemical analysis, which was also used for molecular work (“stem systemic”). The stem sections from five plants were bulked together to provide approximately 0.5 g total per replicate. Leaf sampling of the third unfurled leaf, which was 1 cm above the inoculation point, was the same across all treatments and is described in experiment 1, except that leaf tissue was pooled from five plants per replicate instead of three (**Table 2**). The same pooled leaf tissue was split 1:3 for biochemical and molecular analysis. Exact inoculum concentrations were 1.3 × 10^9^ cfu/ml of Psa, and 1.3 × 10^9^ cfu/ml of Pfm.

Disease assessments were made, on separate plants to those used for biochemical and molecular analyses, 14, 21 and 28 dpi (**Table 2**).

### Hormonal Measurements

#### Sample Preparation

For each biological replicate of pooled frozen stem pieces or leaves that had been pre-ground in liquid nitrogen in a mortar and pestle, a 100 mg sample (fresh weight, FW) was weighed into a 2 mL Eppendorf tube on dry ice and extracted with 400 μL of 10:89:1 methanol:water:acetic acid to which internal standards had been added (25 ng each of [^2^H_4_] SA, [^2^H_5_] JA, [^2^H_6_] ABA, purchased from OlChemim, Czechia). The samples were shaken for 30 min at 4°C, extracted overnight at -20°C and then centrifuged at 13,000 *g* for 10 min at 4°C. The supernatant was removed and the pellet re-extracted with 400 μl of 10:89:1 methanol:water:acetic acid for 60 min at 4°C. Following centrifugation at 13,000 *g* for 10 min at 4°C the supernatants were combined and a 200 μl aliquot transferred to an autosampler vial for analysis by liquid chromatography-mass spectrophotometry (LC-MS).

#### LC-MS Analysis

LC-MS/MS experiments were performed on a 5500 QTrap triple quadrupole/linear ion trap (QqLIT) mass spectrometer equipped with a TurboIon-SprayTM interface (AB Sciex, Concord, ON, Canada) coupled to an Ultimate 3000 UHPLC (Dionex, Sunnyvale, CA, United States).

Plant hormones were separated on a Poroshell 120 SB-C18 2.7 μm 2.1 mm × 150 mm ID column (Agilent Technologies, Santa Clara, CA, United States) maintained at 60°C. Solvents were (A) water + 0.1% formic acid and (B) acetonitrile + 0.1% formic acid and the flow rate was 400 μL min^-1^. The initial mobile phase, 0% B was held for 5 min before ramping linearly to 16% B at 8 min, then to 100% B at 16 min and holding at 100% B until 23 min before resetting to the original conditions. Injection size was 5 μL. MS data were acquired in the negative mode using a Multiple Reaction Monitoring (MRM) method. The transitions monitored (Q1 and Q3) are listed in **Table 3**. Other operating parameters were as follows: dwell time, 50 ms; ionspray voltage, -4500 V; temperature, 600°C; curtain gas, 45 psi; ion source gas 1, 60 psi; ion source gas 2, 60 psi. The Limit of Detection (LoD) was defined as 5× signal to noise.

**Table 3:** Multiple reaction monitoring transitions used for plant hormone analysis.

Q1^a^	Q3^a^	RT^a^	Compound^b^	IS^a^	DP^a^	EP^a^	CE^a^	CXP^a^
141	97	10.5	[^2^H_4_] SA		–35	–10	–30	–15
137	93	10.6	SA	[^2^H_4_] SA	–47	–13	–22	–8
137	65	10.6	SA		–47	–4	–40	–6
269	159	11.7	[^2^H_6_] ABA		–25	–10	–17	–15
263	153	11.8	ABA	[^2^H_6_] ABA	–60	–5	–15	–8
214	62	12.5	[^2^H_5_] JA		–60	–6	–24	–9
209	59	12.5	JA	[^2^H_5_] JA	–60	–6	–24	–9
211	59	13.2	DHJA	[^2^H_5_] JA	–100	–3	–20	–5
322	130	13.7	JA-Ile	[^2^H_5_] JA	–25	–10	–32	–15

*^a^Optimised Q1 and Q3 transitions, retention time (RT), internal standard used for quantification (IS), declustering potential (DP), entrance potential (EP), collision energy (CE) and collision cell exit potential (CXP) for each of the analyzed acidic plant hormones. ^b^SA, salicylic acid; ABA, abscisic acid, JA, jasmonic acid; DHJA, dihydrojasmonic acid; JA-Ile, jasmonic acid isoleucine.*

All data were analyzed and processed using Analyst version 1.6.2 and MultiQuant version 3.0 software packages. Concentrations were calculated on the basis of the peak area for the endogenous compounds relative to those determined for the internal standards.

### RNA Extraction Protocol

A modified version of the López-Gómez and Gómez-Lim (1992) method, developed for extracting RNA from samples with high polysaccharide content, was used to extract RNA from kiwifruit. Modifications included using 25-fold lower sample weights and liquid volumes to enable extraction in Eppendorf tubes, grinding the leaf tissue directly in polyvinylpolypyrrolidone before addition of the lysis buffer and mercaptoethanol, using chloroform rather than chloroform/isoamyl alcohol, and precipitating RNA in absolute ethanol for 1 h at -20°C instead of overnight (J. Bowen and J. Yu, PFR, unpublished data).

RNA concentration was measured using a NanoDrop 200c spectrophotometer (Thermo Scientific, United States) and quality assessed on a 1% (w/v) agarose gel in 0.5× Tris/borate/EDTA (TBE) buffer, pH 7.5.

### Genomic DNA (gDNA) Removal and cDNA Synthesis

RNA extracted from the leaf tissue was treated with a DNA removal kit – Ambion DNAse Turbo^TM^ (Thermo Fisher Scientific, Auckland, New Zealand), used according to manufacturer’s instructions, to remove any contaminant gDNA. DNAse-treated RNA samples were checked by PCR to confirm that there was no gDNA contamination.

cDNA synthesis, using 2 μg RNA per single positive reverse transcriptase reaction for each sample, was performed according to manufacturer instructions using a Bio Rad Tetra cDNA kit (Bioline, Auckland, New Zealand). Samples were then treated with Invitrogen RNAse H (Thermo Fisher Scientific, Auckland, New Zealand).

### Quantitative PCR (qPCR)

qPCR was performed in triplicate on the samples, in a 10 μL reaction volume containing 1 μL of cDNA (diluted 25-fold in water), 1 μL each of forward and reverse primers (10 μM), and 5 μL of Light Cycler 480 SYBR Green 1 Master Mix (Roche Diagnostics GmbH, Mannheim, Germany, Product No. 04 887 352 001). Primers for reference genes (RG) and genes of interest (GoI) (**Table 4**) were designed using Primer3 software (The Whitehead institute, Cambridge, MA, United States) and were synthesized by Invitrogen (Auckland, New Zealand), except for PR1 and PAL, which came from the work of Cellini et al. (2014). Selection of RGs was based on stability of expression in other qPCR studies on kiwifruit (Wurms et al., 2011; Petriccione et al., 2015b). After testing eight different RG, the two RG that were most stably expressed under the conditions of each experiment [actin and elongation factor (EF) for Exp 1, and EF and 40s ribosomal protein (40s) for exp 2] were used for normalization. A gene expression normalization factor (N), calculated using geNorm software v3.4 (Vandesompele et al., 2002) for each sample based on the geometric mean of the RG, was used for the calculation of relative expression of each GoI. The basal transcript level in untreated plants was then used as a further reference point for all calculations and is referred to with the value 1. Choice of GoIs was based upon their putative involvement in kiwifruit resistance against other important pests and diseases (Wurms, 2005; Reglinski et al., 2010; Wurms et al., 2011; Hill et al., 2015), as well as preliminary qPCR studies of kiwifruit responses to Psa (Wurms et al., 2013; Cellini et al., 2014), and a next generation sequencing study of the ‘Hort16A’/Psa interaction (A. Allan, PFR, New Zealand, unpublished data). Published studies of commonly used markers of hormonal pathways (Suza and Staswick, 2008; Ding et al., 2009; Pieterse et al., 2009, 2012; Sanchez-Vallet et al., 2012; Wasternack and Hause, 2013; Meesters et al., 2014), and defense responses of other plants, most often *Arabidopsis thaliana*, to *Pseudomonas syringae* were also used to select GoI (Lorenzo et al., 2004; Pré et al., 2008; Liu et al., 2009). NCBI reference sequences obtained from other plants were used to perform nucleotide BLASTX against the extensive PFR database of kiwifruit expressed sequence tags, assembled into putative genes and genome scaffolds (Crowhurst et al., 2008). Contig sequences with highest bit score and lowest *e*-value were used to identify kiwifruit sequences with the highest similarly to the query sequence. It should, however, be noted that in cases where roles are based largely on functional analyses in *Arabidopsis*, their assignment to a similar function in kiwifruit is primarily by sequence homology and therefore only tentative. A range of 16 GoI were initially chosen to represent as many different defense response pathways and temporal stages of defense as possible. These were: genes involved in early stage defense (RPM1 interacting protein 4, RIN4); markers of the phenyl propanoid pathway that produces antimicrobial secondary metabolites (Phenylalanine ammonia lyase, PAL; Naringenin-chalcone synthase 2, CHS; Cinnamyl alcohol dehydrogenase, CAD); SA pathway markers (WRKY 40 plant transcription factor, WRKY 40; Glucan endo-1,3-beta-glucosidase, β-1,3-glucosidase; Pathogenesis-related protein family 1, PR1; Non-expressor of PR proteins, NPR1; PR family 6 – proteinase inhibitor, PR6); JA pathway markers [MYC2, APETALA2 Ethylene responsive factor 2 (AP2 ERF2), Jasmonate resistant 1 (JAR1); Jasmonoyl-isoleucine-12-hydrolase (JIH1), Lipoxygenase 2 (LOX2)]; and ABA pathway markers (Abscisic acid deficient 1, ABA1; Responsive to dehydration 22, RD22). However, only six genes (**Table 4**) that showed statistically significant treatment effects, and/or expression levels exceeding the commonly used bioinformatic cut-off of ±2-fold changes in differential expression (Mosqueira et al., 2012; Meimoun et al., 2014; Damian-Zamacona et al., 2016) are presented here. Sequence information for the remaining 10 GoI is found in Supplementary Table S1.

**Table 4:** Accession numbers and primer sequences of reference genes (RG) and putative defense-related genes of interest (GoI) used in real-time PCR.

Gene name	Genebank accession number (or Achn number^a^)	Forward primer (5′-3′)	Reverse primer (5′-3′)	Reason for selection and relevant references
RG: Actin	AAA98562	TGCATGAGCGATCAA GTTTCAAG	TGTCCCATGTCTGG TTGATGACT	Ubiquitous protein involved in the formation of filaments of the cytoskeleton and a popular RG for the Psa/kiwifruit interaction (Henty-Ridilla et al., 2013; Petriccione et al., 2015b). Stably expressed in experiment 1.
RG: Elongation Factor, EC 3.6.5.3 (EF)	FG526520	ACAAGCTGGTGAC AATGTGG	CGACCACCTTCATC CTTTGT	Facilitates the elongation steps in protein translation cytoskeleton (Sasikumar et al., 2012). A popular RG for the Psa/kiwifruit interaction (Petriccione et al., 2015b). Stably expressed in both experiments in this study.
RG: 40S Ribosomal Protein (40S)	FG498176	GCAAAGGGATGTG AGGTGAT	CCCCCTGTCTCAGA AGAACA	Involved in synthesis of protein chains (Aitken and Lorsch, 2012). Stable RG in other kiwifruit/pathogen interactions (Wurms et al., 2011). Stably expressed in experiment 2.
GoI: WRKY 40 plant transcription factor (WRKY 40)	Achn 309921 Homolog = At1g80840.1	CTCCAAGCTGCCC TGTTAAG	CTAGTGTCATGCAG CGGCTA	Transcription factor (TF) in the SA pathway which regulates expression of defense genes (Xu et al., 2006; Schoen et al., 2013).
GoI: Glucan endo-1,3-beta-glucosidase EC 3.2.1.39 (β-1,3-glucosidase)	FG455092	TTGGTTCAACATGTCA AAGGAG	TAGGCTGCTTGTTG GGAAAG	Thought to convert preformed inert phytoanticipins (synthesized via the PPP) into their corresponding toxic aglycones (Morant et al., 2008). Involved in other kiwifruit pest/pathogen interactions (Wurms et al., 2011; Hill et al., 2015).
GoI: Pathogenesis-related protein family 1 (PR1)	FG499230	GCCCCCGGTAAG GTTTGT	CGAACCAAGACCCA CTATTGC	Most commonly used marker of SA pathway (Pieterse et al., 2012). Up-regulated by SA-elicitors which decrease Psa infection (Cellini et al., 2014).
GoI: Pathogenesis-related protein family 6 – proteinase inhibitor (PR6)	Achn 075161 Homolog = At5g43580.1	GCCGAAGAGACGAT TGAGAG	AGGGACGCACGTAA CAACAT	PR protein, SA pathway marker, and a proteinase inhibitor (Van Loon et al., 2006).
GoI: APETALA2 Ethylene responsive factor 2 (AP2 ERF2)	Achn 033321 Homolog = At5g47220.1	GAAATATGCGGCA GAAATCC	TTCAGCTGGAAAAT TGAGGAG	Ethylene responsive TF in the JA pathway – JA and ethylene interact to activate plant defensins (Pré et al., 2008).
GoI: Jasmonoyl-isoleucine-12-hydrolase (JIH1)	Achn 018511 Homolog = At3g48520.1	CGGCTCTGATCTG GTTTTTC	CCTCATGCTCTCAC TCAACG	A negative JA pathway marker because JIH1 degrades JA-Ile, the bioactive form of JA, in the JA pathway (Woldemariam et al., 2012).

*The primer sequence for PR1 was obtained from Cellini et al. (2014) and all others were designed in-house. ^a^Achn numbers identify individual the gene models extracted from the *Actinidia chinensis* ‘Honyang’ whole genome shotgun (WGS) project described by Huang et al. (2013), which has the project accession AONS00000000. Nucleotide and amino acid sequences for each model can be found using the Achn numbers at the following web site, which houses the genome http://bioinfo.bti.cornell.edu/cgi-bin/kiwi/home.cgi.*

The relative quantification thermal cycling conditions were: denaturation at 95°C for 10 min, followed by 45 cycles of 10 s denaturation at 95°C, 5 s annealing at a different optimized temperature between 55 and 60°C for each primer set, and 20 s extension at 72°C. Inter-run variability was controlled by including a complete set of treatments on each plate, but a separate run for each biological replicate (i.e., 3 runs/primer set, which were then averaged). Melting curve analysis (60–95°C at 1°C increments with 5 s between each step) was performed after the final qPCR cycle to validate amplicon specificity. Non-template controls were also included to assess the purity of the reagents.

The PCR amplification efficiencies of the primers were determined by creating standard curves based on a dilution series (range = 50–500,000,000 copies/μl) of reference cDNA samples of known concentrations, and only primer pairs with efficiencies of 80% or greater were used in these experiments.

### Statistical Analyses

Results were analysed by analysis of variance (ANOVA) (*P* < 0.05), with data log transformed when necessary to satisfy the assumptions of ANOVA (normal distribution and homogeneity of variances), and means separation by Fisher’s Least Significant Difference (LSD) (*P* < 0.05), using GenStat 17th edition. Disease assessment data from Exp 1 and 2 were analyzed using a repeated measures model, hormone data using a randomised block design (RBD) for Exp 1 and a split-plot design for Exp 2, and qPCR data using a RBD for Exp 1, a RBD for Exp 2 leaves and a nested design for Exp 2 stems The qPCR data for leaf and stem tissue in Exp 2 were analyzed separately because different baselines were used for normalization calculations.

## Results

### Disease Assessments

**FIGURE 1:**
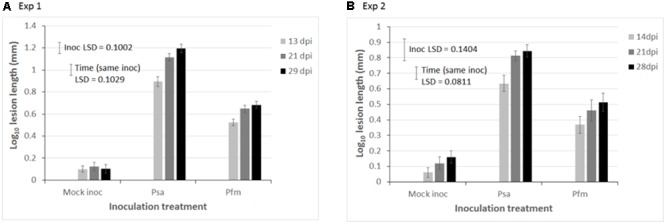
Mean lesion length (mm) on stems of glasshouse grown, tissue cultured ‘Hayward’ plantlets 13–29 days post inoculation (dpi) with water (mock inoculation), or 10^9^ colony forming units (cfu)/ml suspension of *Pseudomonas syringae* pv. *actinidiae* (Psa), or *Pseudomonas syringae* pv. *actinidifoliorum* (Pfm) in **(A)** Experiment (Exp) 1, and **(B)** Exp 2. Data in both experiments were analyzed using a repeated measures experimental model, and a log_10_ transformation was necessary to satisfy the assumptions of ANOVA (normal distribution and homogeneity of variances). Error bars indicate the standard error of the mean (SEM), where *n* = 24 replicate plants in Exp 1 and 10 replicates in Exp 2. Fisher’s Least Significant Difference (LSD) (*P* < 0.05) was used for individual means comparisons. The “Inoc LSD” is used comparing differences between inoculum treatments (Mock inoc vs. Psa vs. Pfm), whilst the “Time (same inoc) LSD” allows for comparisons between different dpi for the same inoculum treatment. The *Y*-axis scale is different in each experiment.

**FIGURE 2:**
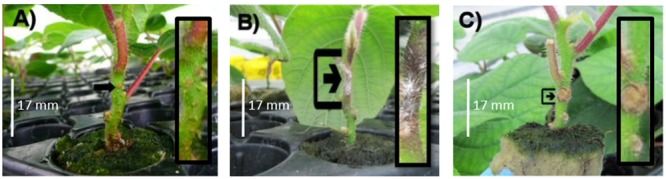
Lesion appearance on stems of glasshouse grown, tissue cultured ‘Hayward’ plantlets 29 days post inoculation (dpi) with **(A)** water (mock inoculation); or a 10^9^ cfu/ml suspension of **(B)** *Pseudomonas syringae* pv. *actinidiae* (Psa); or **(C)** *Pseudomonas syringae* pv. *actinidifoliorum* (Pfm). Insets show close-ups of inoculation points.

Data in both experiments required a log_10_ transformation to satisfy requirements of ANOVA (normal distribution and homogeneity of variances). Lesion length on stems, was significantly greater in both Exp 1 (ANOVA *P* < 0.001, LSD = 0.1002, **Figure 1A**) and Exp 2 (*P* < 0.001, LSD = 0.1404, **Figure 1B**) when kiwifruit plantlets were inoculated with Psa compared with Pfm, irrespective of time (dpi). The difference between Psa and Pfm lesions was greater in Exp 1 than in Exp 2 (**Figure 1**), and lesion size in Psa- and Pfm-inoculated plants significantly increased over time in both experiments (*P* < 0.001 for both experiments, LSD = 0.1209 for Exp 1 and 0.0811 for Exp 2, **Figure 1**). Although lesion length does not necessarily correspond with bacterial titre, the lesion length bioassay provides a good indication of differences in host resistance, as bioassay results correlate well with actual observations of resistance in the field (Datson et al., 2015; Hoyte et al., 2015; Nardozza et al., 2015). As anticipated, Psa symptoms were significantly more pronounced. Lesion development in the mock inoculation consisted only of a small wound at the site of inoculation, which was associated with the stab inoculation method (**Figure 2A**). Pfm lesions had a woodier, less water-soaked appearance than lesions associated with Psa (**Figures 2B,C**).

### Phytohormone Measurements

Data did not require transformation. Phytohormone measurements were consistently lower in Exp 1 than Exp 2, with measurements for JA and SA all below the LoD in Exp 1, except for the JA measurement from Psa-inoculated leaves, 24 hpi, and the SA measurement from Pfm-inoculated leaves, 24 hpi, hence data are not shown. The higher LoDs observed for Exp 1 than Exp 2 were primarily due to differences in instrument sensitivity, which occurred between the two analyses.

Jasmonate isoleucine (JA-Ile) results for both experiments are presented in **Supplementary Figure S1**, because none of the main treatment effects (inoculation treatment and hpi in Exp 1, and inoculation treatment and tissue type in Exp 2), or the interactions between these effects, were statistically significant. One trend worth noting, however, was that JA-Ile concentrations in inoculated leaves were consistently higher at 48 hpi (mean = 6.7 ng/g) than at 24 hpi (mean = 3.3 ng/g) in Exp 1 (LSD = 4.9 ng/g).

**FIGURE 3:**
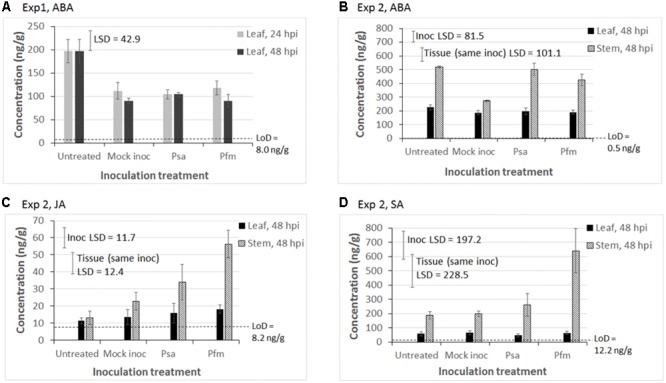
Mean phytohormone concentrations (ng/g of fresh weight) in tissue cultured ‘Hayward’ plantlets of abscisic acid (ABA) in **(A)** Experiment (Exp) 1 and **(B)** Exp 2; **(C)** jasmonic acid (JA) in Exp 2; and **(D)** salicylic acid (SA) in Exp 2. In Exp 1, leaf tissue was sampled 24 and 48 hours post inoculation (hpi) with water (Mock inoc), or 10^9^ cfu/ml suspension of *Pseudomonas syringae* pv. *actinidiae* (Psa), or *Pseudomonas syringae* pv. *actinidifoliorum* (Pfm). Untreated plants were also sampled to obtain a baseline phytohormone concentration. In Exp 2, leaf and stem tissue was sampled 48 hpi. Measurements below the limit of detection (LoD) are considered to be noise. Error bars indicate the standard error of the mean (SEM), where *n* = 3 biological replicates, with each replicate comprising tissue pooled from 3 to 5 plants. Fisher’s Least Significant Difference (LSD) bars enable means comparisons. In Exp 2, the “Inoc LSD” bar is used to compare means between different inoculation treatments and the “Tissue (same inoc) LSD” bar is used to compare means in leaf versus stem tissue, but only for the same inoculation treatment. The *Y*-axis concentration scale is different for each phytohormone and each experiment.

In regards to ABA concentrations, the only statistically significant effect in Exp 1 was that ABA concentrations were higher in untreated leaves than in all other inoculation treatments, irrespective of hpi (*P* < 0.001, LSD = 42.9 ng/g) (**Figure 3A**). Although ABA levels were fractionally higher in untreated leaves (mean = 225.9 ng/g) versus inoculated leaves (mean = 192.3 ng/g) in Exp 2, this difference was not significant (LSD = 81.5 ng/g, **Figure 3B**). The most important effects were that mock inoculation (wounding) was associated with a lower ABA concentration in stems (*P* = 0.029, LSD = 81.5 ng/g), and that ABA concentrations were significantly greater in stems than in leaves irrespective of inoculation treatment (*P* < 0.001, LSD = 101 ng/g, **Figure 3B**).

Similar to the trend observed in Exp 1 (data not shown), JA concentrations in leaves in Exp 2 were low and close to the LoD, and there was no significant effect of inoculation treatment (**Figure 3C**). In contrast, significantly higher JA concentrations were measured in stems than in leaves (*P* < 0.001, LSD = 12.4 ng/g, **Figure 3C**), especially in those stems inoculated with Psa and Pfm, and to a lesser extent by wounding (*P* = 0.009, LSD = 11.7 ng/g, **Figure 3C**).

SA was largely undetectable in leaf tissue in Exp 1, except for Pfm inoculation, 24 hpi (data not shown), and concentrations in leaf tissue in Exp 2 were similar to those in Exp 1. SA concentrations were significantly higher in stem versus leaf tissue, irrespective of inoculation treatment (*P* < 0.001, LSD = 228.5 ng/g, **Figure 3D**). Pfm inoculation induced approximately threefold higher SA in the stem than all other inoculation treatments (*P* = 0.04, LSD = 197.2 ng/g, **Figure 3D**).

### qPCR Measurements

**FIGURE 4:**
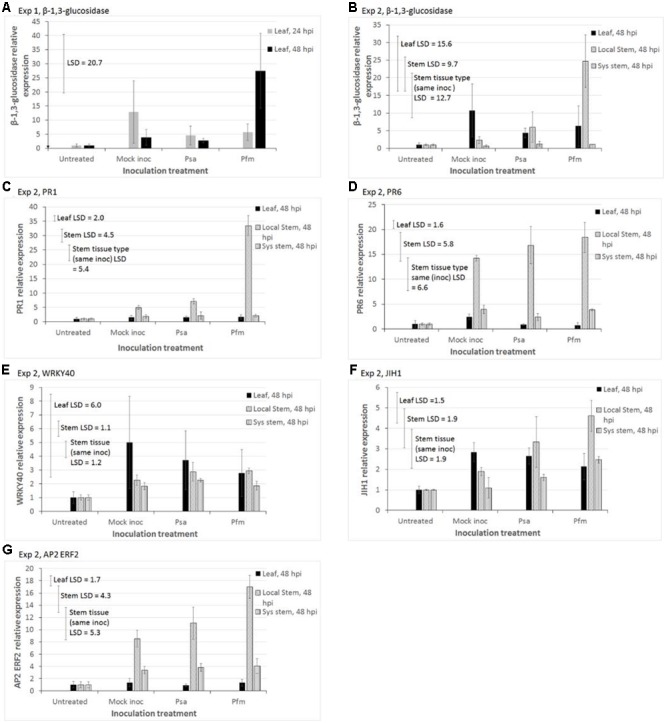
Quantitative polymerase chain reaction (qPCR) measurements of relative expression of putative defense transcripts in two experiments of glasshouse-grown leaves of *Actinidia deliciosa* ‘Hayward’ plantlets. In Exp 1, the leaf above the inoculation point was sampled 24 or 48 hours after stab inoculation (hpi) of the stem with water (Mock inoc), or a 10^9^ cfu/ml suspension of *Pseudomonas syringae* pv. *actinidiae* (Psa), or *Pseudomonas syringae* pv. *actinidifoliorum* (Pfm). In Exp 2 tissue was sampled 48 hpi from three sites – the leaf immediately above the inoculation wound (IW) on the stem (leaf), stem physically surrounding around the IW (local stem), and systemic stem tissue further removed by 1.5 cm from the IW (sys stem). Expression of genes of interest: **(A)** glucan endo-1,3-β-glucosidase (β-1,3-glucosidase), Exp 1; **(B)** β-1,3-glucosidase, Exp 2; **(C)** Pathogenesis-related protein family 1 (PR1), Exp 2; **(D)** PR6, Exp 2; **(E)** WRKY40 transcription factor (WRKY40), Exp 2; **(F)** jasmonoyl-isoleucine-12-hydrolase (JIH1), Exp 2; **(G)** APETALA2/Ethylene response factor 2 transcription factor (AP2 ERF2), Exp 2. Reference genes used for normalization in Exp 1 were actin and elongation factor, whilst elongation factor and 40s protein were used in Exp 2. The basal level of expression in untreated tissue was assigned a value of 1. The *Y*-axis concentration scale is different for each gene of interest and each experiment. Values represent the means ± standard errors of three biological replicates (with each replicate comprising tissue pooled from 3 to 5 plants). Fisher’s Least Significant Difference (LSD) bars enable means comparisons. In Exp 2, the “Leaf LSD” bar is used to compare means between different inoculation treatments for leaf tissue. The “Stem LSD” bar is used to compare means between different inoculation treatments for stem tissue, and the “Stem tissue (same inoc) LSD” bar is used to compare means in local stem versus systemic stem tissue, but only for the same inoculation treatment.

Gene expression results presented here are limited to those results that showed statistically significant treatment effects, and which exceeded the commonly used bioinformatic cut-off of ±2-fold changes in differential expression (Mosqueira et al., 2012; Meimoun et al., 2014; Damian-Zamacona et al., 2016) (**Figure 4**). Results for all other genes, which exceeded the bioinformatic cut-off, are shown in **Supplementary Figure S2**. Results shown in **Figure 4** are for markers of the SA pathway (PR1, β-1,3-glucosidase, PR6, WRKY40), a negative marker of the JA pathway (JIH1), and an ethylene responsive TF in the JA pathway (AP2 ERF2). In both experiments, untreated plant tissue was used as a baseline for qPCR calculations, and was assigned a value of 1, with all other treatments being expressed relative to this value. In Exp 2 there were two separated baselines – one for untreated leaf tissue and one for untreated stems. For this reason, the stem and leaf qPCR data in Exp 2 were analyzed separately, although both sets of results are presented together in **Figure 4**.

In leaf tissue in Exp 1, the main effects of inoculation treatments and time (hpi) and the interaction between these effects were not significant for any of the genes, with the exception of β-1,3-glucosidase (**Figure 4A**), so data are not presented. **Figure 4A** shows that Pfm inoculation resulted in significantly greater β-1,3-glucosidase expression at 48 hpi (mean = 27.5) than at 24 hpi (mean = 5.7, *P* = 0.034, LSD = 20.7). In Exp 2, analysis by ANOVA showed that inoculation treatments did not have any statistically significant effects on any of genes in the leaf tissue. However, individual means comparisons by LSD showed that JIH expression levels in wounded/mock inoculated leaves (mean = 2.8) and Psa-inoculated leaves (mean = 2.7) were significantly greater than those in untreated leaves (mean = 1, LSD = 1.5, **Figure 4F**).

Induction of GoI by Pfm-inoculation in local stem tissue was the strongest response observed in Exp 2 (**Figure 4**). Pfm inoculation was the only treatment to significantly induce β-1,3-glucosidase (*P* = 0.018, LSD = 9.7, **Figure 4B**) and PR1 (*P* < 0.001, LSD = 4.5, **Figure 4C**) in local stem tissue. Wounding/inoculation treatments in stem tissue produced significantly higher levels of PR6 (*P* = 0.013, LSD = 4.7, **Figure 4D**), JIH (*P* = 0.027, LSD = 1.9, **Figure 4F**) and AP2 ERF2 (*P* = 0.003, LSD = 4.3, **Figure 4G**) than in untreated stems, with Pfm-inoculation providing the highest expression in local stems, followed by Psa-inoculation and then mock inoculation.

Wounding/inoculation treatments also lead to increased expression levels of WRKY 40 relative to untreated stems (*P* = 0.028, LSD = 1.1, **Figure 4E**), but the type of inoculum (water, Psa or Pfm) was irrelevant.

**Figure 4** shows that gene expression was always greater (ranging from 2- to 22-fold greater) at the wound site (local stem tissue) than in distal stem tissue, approximately 3 cm from the wound (systemic stem tissue). Comparisons of gene expression in systemic stems versus leaf tissue were more varied – levels in the leaf were mostly greater than in systemic stem tissue (varying from no difference up to 15-fold greater) for WRKY 40, β-1,3-glucosidase and JIH1; approximately the same for PR1; and higher in systemic stems than leaves (by a factor of 1.6- to 3-fold) PR6 and AP2 ERF2 (**Figure 4**).

The most highly expressed genes in both experiments, ranked in descending order, were PR1, β-1,3-glucosidase and PR6, with 32-, 25- and 20-fold increases in local stem tissue, respectively, relative to the untreated control (baseline). Increases relative to wounding (mock inoculation treatment) were 6-, 12- and 1.3-fold, respectively.

## Discussion

The key finding from this study is that ‘Hayward’ kiwifruit are more resistant to stem inoculation with Pfm than with Psa biovar3, and that the enhanced resistance is associated with a stronger, localized SA-based defense response. Differential responses in hormone concentrations occurred only in the stem tissue close the inoculation site and showed that Pfm inoculation induced a threefold increase in SA relative to Psa inoculation and a smaller 1.6-fold induction of JA. Transcript expression showed that Pfm inoculation of the stem, compared with wounding and Psa inoculation, resulted in the greatest elevation of PR1 and PR6 (SA pathway markers), β-1,3-glucosidase (involved in activation of phytoanticipins and a SA pathway marker), AP2 ERF2 transcription factor (an early defense response marker involved in SA/JA cross talk), and JIH1 (an enzyme degrading the active metabolite JA-Ile in the JA pathway) in stem tissue surrounding the inoculum site. These genes have potential as markers of ‘Hayward’ resistance to *Pseudomonas syringae* pathovars and, in particular, PR1 and β-1,3-glucosidase appear to be useful marker candidates for distinguishing the host response to Psa and Pfm.

Results from this study indicate that method of inoculation, choice of sampling tissue and timing of collection are important factors to consider when carrying out biochemical and molecular studies. Phytohormone and gene expression measurements showed that significant results were only observed in localized stem tissue that was collected 48 hpi, and not in leaves that were sampled 24 and 48 hpi. However, a systemic response in leaves and in distal stem tissue might have been observed in this study if sample times of greater than 48 hpi had been used. Method of inoculation also needs to be considered. For example, several proteomic studies showed Psa inoculation induced up-regulation of proteins associated with SA-defense in ‘Hayward’ and ‘Hort16A’ leaves 72 hpi (Petriccione et al., 2013, 2014, 2015a), but the inoculation method consisted of spraying all the leaves with Psa, hence a localized response to leaf inoculation was effectively being measured. Cellini et al. (2014) also measured a localized response in leaf tissue, i.e., up-regulation of SA marker genes 24–72 hpi, because their method of inoculation comprised cutting leaf tips with inoculum-dipped scissors. Stem inoculation was utilized in the current study because resistance levels determined by this bioassay method have shown to correlate well with actual levels of resistance observed in the field (Datson et al., 2015; Hoyte et al., 2015; Nardozza et al., 2015). Further research with these molecular markers should include carrying out a time course, because sampling 24–48 dpi may be adequate for determining localized expression of PR proteins that are activated late during a defense response, as indicated by other studies (Cellini et al., 2014; Petriccione et al., 2014), but may be less suitable for capturing earlier expressed localized defenses, or delayed systemic responses.

In both artificial inoculation experiments of ‘Hayward’ plantlets, disease severity, as indicated by lesion length, increased over time and was always significantly greater with Psa (strain #10627) inoculation of the stem than with Pfm (ICMP strain #19098) inoculation. Originally thought to be different biovars of the same pathogen (Vanneste et al., 2013), Pfm was designated as a separate pathovar from Psa due to pathogenic, phenotypic, genetic and phylogenetic differences (Cunty et al., 2015). Pfm is substantially less aggressive than Psa in that it is only able to cause leaf spots and has not been linked with commercial loss of productivity in the New Zealand kiwifruit industry. In contrast, Psa can additionally cause canker and shoot dieback and is considered to be a major kiwifruit pathogen (Vanneste et al., 2013). It is possible that the increased ability of the host to mount a response to Pfm is largely responsible for its lack of virulence. There are several ways to explain this. One is that Pfm lacks a number of critical pathogen factors to suppress the host response that Psa biovar 3 (bv3) possesses. Genome-based comparisons of Psa and Pfm (McCann et al., 2013) clearly indicate a significant series of effectors present in Psa bv3 that are missing from Pfm that could account for this behavior. On the other hand, there could be Pfm factors such as effectors (of which there are several not present in Psa bv3) and/or PAMPs recognized by kiwifruit that turn on a defense response. Typically when a plant recognizes effectors, it initiates resistance gene-based responses, which have characteristics such as hypersensitive responses that are not seen in the response of kiwifruit to Pfm, which makes this scenario less likely. However, it is important to note that resistance responses to effectors are not invariably linked to obvious hypersensitive responses. The most likely explanation, however, is that one or more of the effectors present in Psa bv3, but absent in Pfm, actively suppresses the response mounted by the host against Pfm and this explains the virulence of Psa bv3 and, by contrast, the lack of virulence of Pfm.

Phytohormone measurements suggest predominance of the SA pathway over the JA pathway, in host defense against the pathovars used in this study, with a greater response induced by Pfm than Psa. Phytohormone measurements in leaves were not significant, except for elevated levels of ABA in untreated versus inoculated leaves in Exp 1, but this result was not observed in Exp 2 leaf tissue. Given that no other hormones responded to inoculation treatments in leaf tissue, the Exp 2 result for ABA is more likely to be correct. In contrast, localized induction of SA in stems was up to threefold higher with Pfm inoculation than with Psa (*P* < 0.001), and a smaller induction (1.6-fold) of JA (*P* < 0.009) was also observed. *P. syringae* is able to stimulate both SA and JA pathways (Pieterse et al., 2012), but resistance to many biotrophic bacterial pathogens, which infect aerial parts of plants, including *Pseudomonas* spp., is mediated via SA-responsive signaling in a number of diverse hosts including rice, tobacco and *Arabidopsis* (Lee et al., 2013; Yang et al., 2016). In support of these findings, Cellini et al. (2014) found that compounds that stimulated the SA pathway (SA or its synthetic analog, acibenzolar-S-methyl ASM) reduced disease caused by Psa on ‘Hayward,’ while methyl-jasmonate or ethylene increased disease development. These results fit the most simplistic model of antagonistic SA/JA cross talk, where up-regulation of one pathway leads to down-regulation of the other. However, SA/JA cross talk appears much more complex than this, because neutral and synergistic interactions have also been recorded (Schenk et al., 2000; Van Wees et al., 2000; Mur et al., 2006). Mur et al. (2006) showed that the outcome of the SA–JA interaction is dependent of the relative concentration of each hormone. In the current study, SA concentration was elevated by Pfm more than JA, and there was no increase in the bioactive form of jasmonate JA-Ile, suggesting that SA/JA antagonism is occurring. The effect of other phytohormones on SA/JA cross talk also needs to be considered (Pieterse et al., 2012). Wounding significantly reduced ABA levels in the local stem tissue in Exp 2 (*P* = 0.029), which was most likely associated with movement of all the treated plants (Mock inoc, Psa and Pfm) into a higher humidity environment after inoculation (Okamoto et al., 2009). This suggests that the plant maintains high internal levels of ABA in the stem in response to the presence of *P. syringae* pathogens, where it may have been acting as a regulator, coordinating stress responses (Bostock et al., 2014; de Zelicourt et al., 2016). Using hormone data alone to draw conclusions must be viewed with caution, as hormone expression is influenced by many factors including tissue type (as shown in the current research), age, external abiotic and biotic factors and circadian rhythms (Zheng et al., 2015). For this reason, the current study also examined gene expression, including mRNA markers of the phytohormone pathways.

Gene expression measurements suggest that host resistance to Pfm is multigenic, localized and predominantly involves the SA rather than the JA pathway, but with a degree of cross talk between SA-dependent and JA/ethylene dependent pathways. Although 16 GoI putative markers were selected to represent as many different pathways and temporal stages in defense as possible, only six genes were significantly affected by inoculation treatment, and exceeded the commonly used bioinformatic cut-off of ±2-fold changes in differential expression (Mosqueira et al., 2012; Meimoun et al., 2014; Damian-Zamacona et al., 2016). Of these 16 genes, 4/5 SA markers were significantly induced (PR1, β-1,3-glucosidase, PR6, WRKY40), and 2/5 of JA markers (AP2 ERF 2, which is known to be involved in SA/JA cross talk, and JIH1 which suppresses the JA pathway). In contrast, RIN4, phenyl propanoid pathway markers (PAL, CHS and CAD) and ABA pathway markers (ABA1 and RD22) all had less than twofold changes in differential expression, which is considered to be the biological equivalent of no change in expression. Expression of PR1, β-1,3-glucosidase, PR6, JIH1 and AP2 ERF2 in stem tissue surrounding the inoculum site was most strongly induced by Pfm inoculation, whilst WRKY40 transcription factor was induced equally in stems by wounding (mock inoculation) and pathovar inoculation. PR1 is the most commonly used marker of the SA pathway (Pieterse et al., 2012). Using the same marker for PR1 as this study, Cellini et al. (2014) showed that PR1 and other SA-pathway markers, PR8 (chitinase) and isochorismate synthase, were more strongly up-regulated in ‘Hayward’ than in ‘Hort16A’ plants treated with the SA-analog, ASM, which correlated with a greater reduction of disease caused by Psa. A recent transcriptome study also showed that pre-treatment of kiwifruit with ASM, leads to a much greater proliferation of defense related sequences associated with the SA pathway upon inoculation with Psa, suggesting that the molecular response is strongly enhanced in ASM-treated plants (Michelotti et al., 2015).

β-1,3-glucosidase in the current study was also most strongly induced by Pfm inoculation. Glucosidases enable the plant to respond immediately to pathogen invasion by converting preformed inert phytoanticipins into their corresponding toxic aglycones by sugar hydrolysis (Osbourn, 1996; Zagrobelny et al., 2004; Morant et al., 2008). In addition to deglycosylation of inactive storage precursors formed via the phenyl propanoid pathway, β-1,3-glucosidases can hydrolyse conjugated plant hormones, thereby altering their bioactivity (Minic, 2008), and enzyme hydrolysis can also produce elicitor compounds. Glucosidases are also considered to be closely related to the PR2 family of enzymes (glucanases) which are often used as markers of the SA-pathway (Thatcher et al., 2005; Minic, 2008). Wurms et al. (2011) showed that β-1,3-glucosidase transcript expression reduced significantly over time (*P* < 0.001), in concert with increased ripe rot disease incidence caused by *Cryptosporiopsis*. Other studies have shown that hydrolysis of fruit and leaf phenolic extracts from ‘Hayward’ and ‘Hort16A’ greatly increases the microbial toxicity of these extracts (Wurms et al., 2003; Wurms, 2005). In addition, hydrolysed phenolics of ‘Hort16A’ were more fungitoxic than those from ‘Hayward,’ which correlated positively with greater resistance of ‘Hort16A’ than ‘Hayward’ to *Botrytis cinerea* and *Sclerotinia sclerotiorum* infections (Reglinski et al., 2002; Wurms et al., 2003; Wurms, 2005). The same β-1,3-glucosidase transcript that was analyzed in the current study has also been implicated in ‘Hort16A’ defense against scale insects (Hill et al., 2015). In support of these molecular and biochemical findings, a proteomic study of ‘Hayward’ leaf colonization by Psa showed elevated expression of two beta-1,3-glucosidase isoforms 3 days after inoculation compared with a water control (Petriccione et al., 2014).

The strongest induction of JIH (a negative marker of the JA pathway) by Pfm, followed by Psa, in the current study provides further weight to the hypothesis that defense against Pfm and Psa primarily involves the SA pathway. JIH1 degrades the active metabolite JA-Ile, with attenuation of the JA-Ile burst (Woldemariam et al., 2012), possibly allowing the plant to tailor the expression of SA-mediated responses.

Pfm was the strongest inducer of AP2 ERF2 (kiwifruit homolog of AtERF2), particularly in local stem tissue, which suggests some degree of cross talk between SA/JA. Some genes from the AP2/ERF family can be used as markers of ethylene/JA induction, and Van der Does et al. (2013) found that they have an important role to play in JA/SA cross talk. Their data showed that SA can inhibit JA signaling by targeting/negatively affecting GCC-box motifs on JA-responsive promoters of the AP2 ERF transcription factor family, inhibiting accumulation of some members but not others, for example ORA59, but not ERF1. As SA concentrations were higher with Pfm than Psa inoculation, the concentrations of the AP2 ERF2 marker in this study do not appear to be adversely affected by SA. Pré et al. (2008) observed that ERF1, but not AtERF2, was able to activate plant defensin expression (a JA defense response). Other evidence of more complex SA/JA cross-talk occurring in kiwifruit comes from the current study, where WRKY40, which is associated with the SA pathway (Xu et al., 2006; Schoen et al., 2013), was shown to be induced equally strongly by wounding and by Psa/Pfm inoculation, yet wounding is considered to be a JA pathway response (Vasyukova and Ozeretskovskaya, 2009; Wasternack and Song, 2017). In addition, activity of PR6 (a SA pathway marker) was highest with Pfm inoculation, but induction by wounding was almost as strong. Cellini et al. (2014) also found that lipoxygenase 2, a commonly used JA pathway marker (Garcia-Marcos et al., 2013; Wasternack and Hause, 2013), was expressed more highly in ‘Hayward’ than ‘Hort16A’ kiwifruit, which are more resistant to Psa, but expression levels were lower and more transient than that of the SA pathway markers, PR1 and ICS. It is highly unlikely that there is complete antagonism between the SA and JA pathways in response to Psa/Pfm infection in kiwifruit, given that numerous studies in *Arabidopsis* have shown that activated signaling pathways are not simply linear, but rather form complex networks where considerable cross talk takes place (Thatcher et al., 2005; Pieterse et al., 2012).

One caveat of transcription studies is that correlations between mRNA expression and induced disease resistance alone do not constitute proof of involvement in defense, as mRNA levels may not necessarily always correlate with changes in protein levels (Gygi et al., 1999; Chen et al., 2002). This is further complicated by factors such as post-translational modification which may result in more or less active versions of proteins, or versions that are more or less susceptible to proteolytic degradation. In addition, responses to stresses such as pathogens are likely to have multiple temporal profiles. Response time at the proteomic level is potentially much faster than the transcriptomic response as sometimes all it requires is a post-translational phosphorylation by an existing protein kinase to change protein activity. Bearing in mind these caveats, a proteomic analysis of the localized ‘Hayward’ leaf response to Psa colonization also showed that SA pathway associated PR proteins – PR1, PR2 (glucanases), glucosidases, PR3 (chitinases), PR4 (chitinases), PR5 (thaumatin like proteins), PR9 (peroxidases) and PR17 were the most up-regulated proteins (Petriccione et al., 2014, 2015a). A similar study in ‘Hort16A’ showed significant up-regulation of PR families 1, 2, 3, 4, 5 and 10 (Petriccione et al., 2013), hence proteomic analyses support the gene expression findings of the current study.

To our knowledge, this is the first report of localized versus systemic kiwifruit transcriptomic and phytohormone responses to closely related *Pseudomonas syringae* pathovars. It suggests that reduced severity of Pfm infection compared with Psa infection is due to a greater ability of kiwifruit to mount defense responses to Pfm. Gaining an understanding of the defense pathways and phytohormones involved in the defense response to Psa can help us to tailor elicitor choice and application to optimize their efficacy. Genes such as PR1 and β-1,3-glucosidase, which proved to be effective markers of resistant phenotype expression in the current study, may be used to screen breeding populations to select for more resistant phenotypes. These strategies are being used to develop an integrated fruit production system that is both more durable and more beneficial to the environment and consumers.

## Author Contributions

KW – designed experiments, set up trials, carried out all molecular work, including primer design and developing qPCR protocols, data analysis, wrote the manuscript. AH – carried out all molecular work and analysis including qPCR troubleshooting, editorial input into manuscript. AAC – set up trials, carried out disease assessments, molecular work including qPCR troubleshooting, editorial input into manuscript. JB – developed and modified protocols for successful RNA extraction thus overcoming a key technical hurdle, editorial input into manuscript. JP – molecular work including qPCR system optimisation, editorial input into manuscript. JT – set up trials, carried out disease assessments, data analysis, editorial input into manuscript. DJ – carried out all chemical protocols including trouble shooting, editorial input into manuscript. JC – developed phytohormone protocols and carried out all chemical work, editorial input into manuscript. TR – experimental design, input into chemical protocols, identification of most suitable phytohormone markers, data interpretation, major editorial input. MW – statistical analysis and editorial input.

## Conflict of Interest Statement

Theauthors declare that the research was conducted in the absence of any commercial or financial relationships that could be construed as a potential conflict of interest.

## Supplementary Material

FIGURE S1 Mean phytohormone concentrations (ng/g of fresh weight) in tissue cultured ‘Hayward’ plantlets of jasmonate isoleucine (Ja-Ile) in **(A)** Experiment (Exp) 1 and **(B)** Exp 2. In Exp 1, leaf tissue was sampled 24 and 48 hours post inoculation (hpi) with water (Mock inoc), or 10^9^ cfu/ml suspension of *Pseudomonas syringae* pv. *actinidiae* (Psa), or *Pseudomonas syringae* pv. *actinidifoliorum* (Pfm). Untreated plants were also sampled to obtain a baseline phytohormone concentration. In Exp 2, leaf and stem tissue was sampled 48 hpi. Measurements below the limit of detection (LoD) are considered to be noise. Error bars indicate the standard error of the mean (SEM), where *n* = 3 biological replicates, with each replicate comprising tissue pooled from 3 to 5 plants. Fisher’s Least Significant Difference (LSD) bars enable means comparisons. In Exp 2, the “Inoc LSD” bar is used to compare means between different inoculation treatments and the “Tissue (same inoc) LSD” bar is used to compare means in leaf versus stem tissue, but only for the same inoculation treatment. The *Y*-axis concentration scale is different for each phytohormone and each experiment.

FIGURE S2 qPCR measurements of relative expression (log_10_ values) of putative defense transcripts in glasshouse-grown leaves of *Actinidia deliciosa* ‘Hayward’ plantlets. The leaf above the inoculation point was sampled 24 or 48 hours after stab inoculation (hpi) of the stem with water (Mock inoc), or a 10^9^ cfu/ml suspension of Psa, or Pfm. Expression of genes of interest: **(A)** Pathogenesis-related protein family 1 (PR1); **(B)** PR6; **(C)** WRKY40 transcription factor (WRKY40); **(D)** jasmonoyl-isoleucine-12-hydrolase (JIH1); and **(E)** APETALA2/Ethylene response factor 2 transcription factor (AP2 ERF2). Reference genes used for normalization were actin and elongation factor. The basal level of expression in untreated tissue was assigned a value of 1. Values represent the means ± standard errors of three biological replicates. Fisher’s Least Significant Difference (LSD) bars enable means comparisons. Genes presented here are those that had more than twofold differential changes in expression relative to untreated tissue, but which did not show statistically significant treatment effects. The *Y*-axis concentration scale is different for each gene of interest.
