# Corrigendum: Phytohormone and Putative Defense Gene Expression Differentiates the Response of ‘Hayward’ Kiwifruit to Psa and Pfm Infections

**DOI:** 10.3389/fpls.2017.02012

**Published:** 2017-11-22

**Authors:** Kirstin V. Wurms, Allan J. Hardaker, Annette Ah Chee, Judith Bowen, Janet Phipps, Joseph Taylor, Dwayne Jensen, Janine Cooney, Mark Wohlers, Tony Reglinski

**Affiliations:** ^1^The New Zealand Institute for Plant & Food Research Limited, Hamilton, New Zealand; ^2^The New Zealand Institute for Plant & Food Research Limited, Auckland, New Zealand

**Keywords:** bacterial canker, defense gene expression, host resistance, phytohormone regulation, plant–pathogen interactions

In the original article, there was an error in the total number of cycles and the timing of cycle steps in the qPCR protocol.

A correction has been made to the Materials and Methods section, under the sub-heading Quantitative PCR (qPCR), paragraph two:

The relative quantification thermal cycling conditions were: denaturation at 95°C for 10 min, followed by 40 cycles of 15 s denaturation at 95°C, 15 s annealing at a different optimized temperature between 55 and 60°C for each primer set, and 20 s extension at 72°C.

The authors apologize for this error and state that this does not change the scientific conclusions of the article in any way.

## Conflict of interest statement

The authors declare that the research was conducted in the absence of any commercial or financial relationships that could be construed as a potential conflict of interest.

