# Supplementary figures and images for: Phytohormone and Putative Defense Gene Expression Differentiates the Response of ‘Hayward’ Kiwifruit to Psa and Pfm Infections
Source: Front Plant Sci. 2017 Aug 4;8:1366. doi: 10.3389/fpls.2017.01366 (PMC5543098; doi:10.3389/fpls.2017.01366)

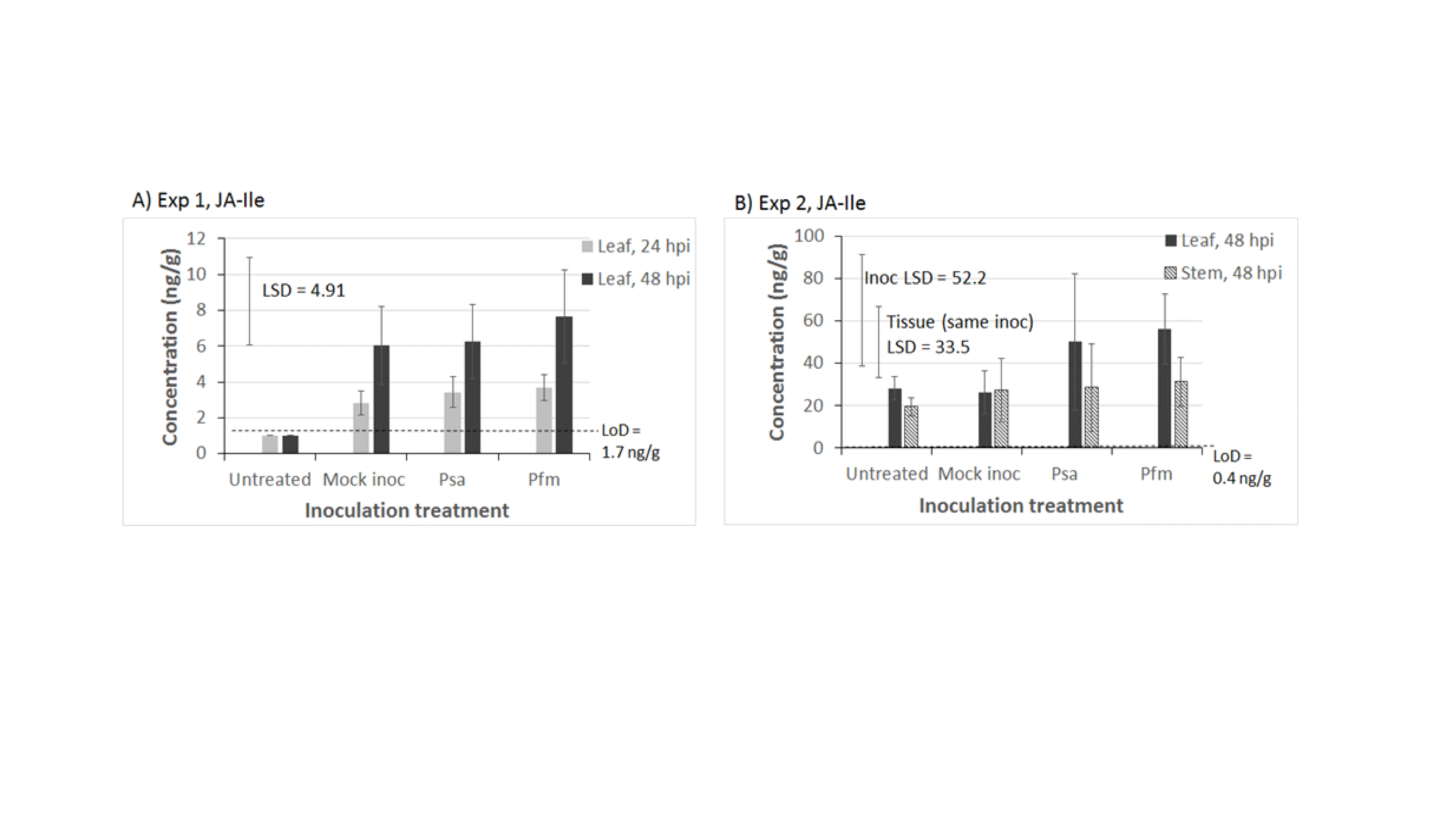

Supplement: FIGURE S1 — Mean phytohormone concentrations (ng/g of fresh weight) in tissue cultured ‘Hayward’ plantlets of jasmonate isoleucine (Ja-Ile) in (A) Experiment (Exp) 1 and (B) Exp 2. In Exp 1, leaf tissue was sampled 24 and 48 hours post inoculation (hpi) with water (Mock inoc), or 109 cfu/ml suspension of Pseudomonas syringae pv. actinidiae (Psa), or Pseudomonas syringae pv. actinidifoliorum (Pfm). Untreated plants were also sampled to obtain a baseline phytohormone concentration. In Exp 2, leaf and stem tissue was sampled 48 hpi. Measurements below the limit of detection (LoD) are considered to be noise. Error bars indicate the standard error of the mean (SEM), where n = 3 biological replicates, with each replicate comprising tissue pooled from 3 to 5 plants. Fisher’s Least Significant Difference (LSD) bars enable means comparisons. In Exp 2, the “Inoc LSD” bar is used to compare means between different inoculation treatments and the “Tissue (same inoc) LSD” bar is used to compare means in leaf versus stem tissue, but only for the same inoculation treatment. The Y-axis concentration scale is different for each phytohormone and each experiment. [file Image_1.TIF]

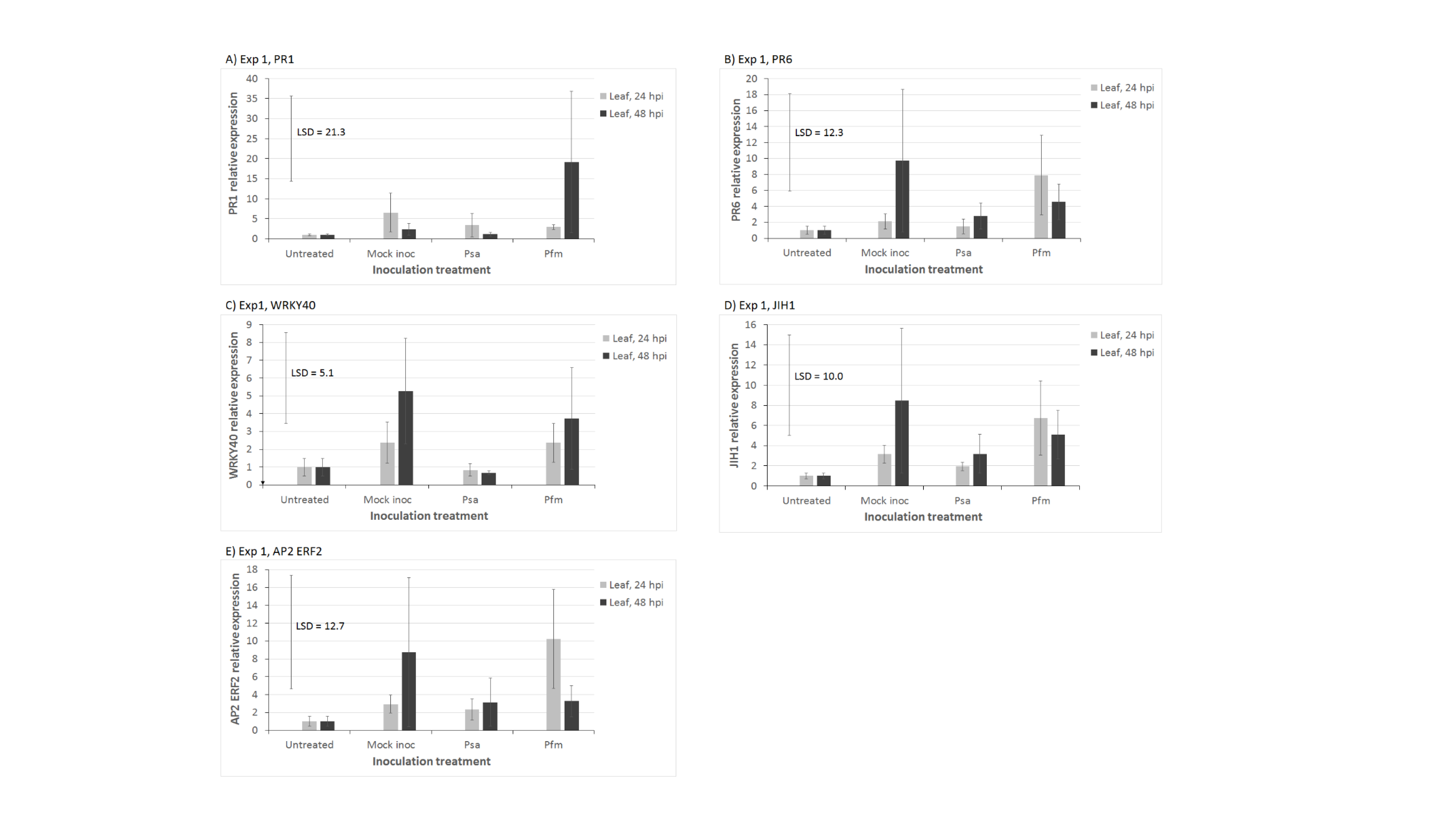

Supplement: FIGURE S2 — qPCR measurements of relative expression (log10 values) of putative defense transcripts in glasshouse-grown leaves of Actinidia deliciosa ‘Hayward’ plantlets. The leaf above the inoculation point was sampled 24 or 48 hours after stab inoculation (hpi) of the stem with water (Mock inoc), or a 109 cfu/ml suspension of Psa, or Pfm. Expression of genes of interest: (A) Pathogenesis-related protein family 1 (PR1); (B) PR6; (C) WRKY40 transcription factor (WRKY40); (D) jasmonoyl-isoleucine-12-hydrolase (JIH1); and (E) APETALA2/Ethylene response factor 2 transcription factor (AP2 ERF2). Reference genes used for normalization were actin and elongation factor. The basal level of expression in untreated tissue was assigned a value of 1. Values represent the means ± standard errors of three biological replicates. Fisher’s Least Significant Difference (LSD) bars enable means comparisons. Genes presented here are those that had more than twofold differential changes in expression relative to untreated tissue, but which did not show statistically significant treatment effects. The Y-axis concentration scale is different for each gene of interest. [file Image_2.TIF]
