# Supplementary material for: Phytohormone and Putative Defense Gene Expression Differentiates the Response of ‘Hayward’ Kiwifruit to Psa and Pfm Infections
Source: Front Plant Sci. 2017 Aug 4;8:1366. doi: 10.3389/fpls.2017.01366 (PMC5543098; doi:10.3389/fpls.2017.01366)
Supplement: Supplementary file 3 [file Table_1.docx]

**Supplementary Table 1: Accession numbers and primer sequences of putative defence-related genes of interest (GoI) used in real-time PCR that did not show statistically significant results and/or which had less than ± 2-fold changes in differential expression.**

| **Gene name** | **Genebank accession number (or Achn number^a^)** | **Forward primer (5′- 3′)** | **Reverse primer (5′- 3′)** | **Reason for selection and relevant references** |
| --- | --- | --- | --- | --- |
| GoI: RPM interacting protein 4 (RIN4) | NP_189143 | CTTTGAGAAAGCACGGAAGG | TGGTTCTTCTGGTTCGGTTC | Early stage defence – involved in the guard cell response against bacterial pathogens (Liu et al., 2009). Significantly upregulated by SA-elicited, Psa-infected kiwifruit (Wurms et al., 2013). |
| GoI: Phenylalanine ammonia lyase, EC 4.3.1.5 (PAL) | Achn 060261 Homologue = At2g37040.1 | CGGAGCAACACAACCAAGA | CCTGACATAGTGCGACTACATAG | Key regulatory enzyme of the phenylpropanoid pathway (PPP), which creates antimicrobial secondary metabolites (Naoumkina et al., 2010). Up-regulated by SA-elicitors which decrease Psa infection (Cellini et al., 2014). |
| GoI: Naringenin-chalcone synthase 2, EC 2.3.1.74 (CHS) | NP_196897 | GTCAAGCGCCTCATGATGTA | ATCACTGGGTCCACGAAAAG | Key regulatory enzyme of the PPP, which creates antimicrobial secondary metabolites (Naoumkina et al., 2010). |
| GoI: Cinnamyl alcohol dehydrogenase (CAD) | Achn 239241 Homologue = At4g37980.1 | TTCAAAGCTGGGGACAGAGT | AGGGGTCCCATCAAAGTAGG | Key enzyme in latter stages of lignin biosynthesis (Trabucco et al., 2013). Lignin forms a physical barrier to invaders and the lignin pathway is a side branch of the PPP. |
| GoI: Non-expressor of PR proteins (NPR1) | Not released.  Homologue =  XM 002281439.4 | AGTTCCCATTGTCCGTCATC | AAGAAACGCTTCCCCAATTC | Along with PR1, NPR1 is commonly used as a marker of the SA pathway, and acts as a transcriptional co-activator of PR gene expression (Pieterse et al., 2009). |
| GoI: MYC2 | Achn 136071 Homologue = KU892080.1 | GCTGTGGTGCCCAATATCTC | GTTTGCCTCCAAATCGAGTG | TF in the JA pathway, leads to production of vegetative storage proteins (VSPs) which are often associated with the wound response and defence against insect herbivores (Lorenzo et al., 2004). |
| GoI: Jasmonate resistant 1 (JAR1) | Not released.  Homologue =  XM 017380367.1 | TTCTACATTTGCCCACAGCA | TTTGGCTTGAGCAGTTTTGA | One of the most commonly used markers of the JA pathway, which synthesizes the most bioactive form of the JA hormone jasmonate isoleucine (JA-Ile) (Suza and Staswick, 2008; Meesters et al., 2014). |
| GoI: Lipoxygenase 2 (LOX2) | Achn 123621 Homologue = At3g45140.1 | CAGAGCTTGATCCCAAGACA | CAGGATGGCTCCAATTTCAC | Commonly-used marker of the JA pathway, (Garcia-Marcos et al., 2013; Wasternack and Hause, 2013). |
| GoI: Abscisic acid deficient 1 (ABA1) | Achn 013171 Homologue = At5g67030.1 | GTAGATGGTCCCGCTGGTAA | TTTCCCCACGTAAAAATTGG | Involved in abscisic acid (ABA) biosynthesis. Commonly used marker of the ABA pathway (Ding et al., 2009; Sanchez-Vallet et al., 2012). |
| GoI: Responsive to dehydration 22 (RD22) | Not released.  Homologue =  XM 011021370.1 | GACCTCAAAAAGGGCACAAA | AGTCGGGCTTCACAGAAAAG | A reliable marker of the early stage ABA response, associated with ABA-mediated drought tolerance (Ding et al., 2009; Matus et al., 2014). |

^a^Achn numbers identify individual the gene models extracted from the *Actinidia chinensis* ‘ Honyang’ whole genome shotgun (WGS) project described by Huang et al. (2013), which has the project accession AONS00000000. Nucleotide and amino acid sequences for each model can be found using the Achn numbers at the following web site, which houses the genome <http://bioinfo.bti.cornell.edu/cgi-bin/kiwi/home.cgi>

**REFERENCES**

Cellini, A., Fiorentini, L., Buriani, G., Yu, J., Donati, I., Cornish, D.A., Novak, B., Costa, G., Vanneste, J.L., and Spinelli, F. (2014). Elicitors of the salicylic acid pathway reduce incidence of bacterial canker of kiwifruit caused by *Pseudomonas syringae* pv. *actinidiae*. *Ann. Appl. Biol.* 165**,** 441-453. doi: 10.1111/aab.12150

Ding, Z.H., Li, S.M., An, X.L., Liu, X.J., Qin, H.M., and Wang, D. (2009). Transgenic expression of MYB15 confers enhanced sensitivity to abscisic acid and improved drought tolerance in *Arabidopsis thaliana*. *J. Genet. Genomics* 36**,** 17-29.

Garcia-Marcos, A., Pacheco, R., Manzano, A., Aguilar, E., and Tenllado, F. (2013). Oxylipin biosynthesis genes positively regulate programmed cell death during compatible infections with the synergistic pair potato virus X-potato virus Y and tomato spotted wilt virus. *J. Virol.* 87**,** 5769-5783. doi: 10.1128/jvi.03573-12

Huang, S.X., Ding, J., Deng, D.J., Tang, W., Sun, H.H., Liu, D.Y., Zhang, L., Niu, X.L., Zhang, X., Meng, M., Yu, J.D., Liu, J., Han, Y., Shi, W., Zhang, D.F., Cao, S.Q., Wei, Z.J., Cui, Y.L., Xia, Y.H., Zeng, H.P., Bao, K., Lin, L., Min, Y., Zhang, H., Miao, M., Tang, X.F., Zhu, Y.Y., Sui, Y., Li, G.W., Sun, H.J., Yue, J.Y., Sun, J.Q., Liu, F.F., Zhou, L.Q., Lei, L., Zheng, X.Q., Liu, M., Huang, L., Song, J., Xu, C.H., Li, J.W., Ye, K.Y., Zhong, S.L., Lu, B.R., He, G.H., Xiao, F.M., Wang, H.L., Zheng, H.K., Fei, Z.J., and Liu, Y.S. (2013). Draft genome of the kiwifruit *Actinidia chinensis*. *Nature Communications* 4**,** 9. doi: 10.1038/ncomms3640

Liu, J., Elmore, J.M., Fuglsang, A.T., Palmgren, M.G., Staskawicz, B.J., and Coaker, G. (2009). RIN4 functions with plasma membrane H+-ATPases to regulate stomatal apertures during pathogen attack. *PLoS Biol.* 7. doi: 10.1371/journal.pbio.1000139

Lorenzo, O., Chico, J.M., Sanchez-Serrano, J.J., and Solano, R. (2004). Jasmonate-insensitive1 encodes a MYC transcription factor essential to discriminate between different jasmonate-regulated defense responses in *Arabidopsis*. *Plant Cell* 16**,** 1938-1950. doi: 10.1105/tpc.022319

Matus, J.T., Aquea, F., Espinoza, C., Vega, A., Cavallini, E., Dal Santo, S., Canon, P., De La Guardia, A.R.H., Serrano, J., Tornielli, G.B., and Arce-Johnson, P. (2014). Inspection of the grapevine BURP superfamily highlights an expansion of RD22 genes with distinctive expression features in berry development and ABA-mediated stress responses. *PLoS ONE* 9. doi: 10.1371/journal.pone.0110372

Meesters, C., Monig, T., Oeljeklaus, J., Krahn, D., Westfall, C.S., Hause, B., Jez, J.M., Kaiser, M., and Kombrink, E. (2014). A chemical inhibitor of jasmonate signaling targets JAR1 in *Arabidopsis thaliana*. *Nat. Chem. Biol.* 10**,** 830-+. doi: 10.1038/nchembio.1591

Naoumkina, M.A., Zhao, Q., Gallego-Giraldo, L., Dai, X., Zhao, P.X., and Dixon, R.A. (2010). Genome-wide analysis of phenylpropanoid defence pathways. *Mol. Plant Pathol.* 11**,** 829-846. doi: 10.1111/j.1364-3703.2010.00648.x

Pieterse, C.M.J., Leon-Reyes, A., Van Der Ent, S., and Van Wees, S.C.M. (2009). Networking by small-molecule hormones in plant immunity. *Nat. Chem. Biol.* 5**,** 308-316. doi: 10.1038/nchembio.164

Sanchez-Vallet, A., Lopez, G., Ramos, B., Delgado-Cerezo, M., Riviere, M.P., Llorente, F., Fernandez, P.V., Miedes, E., Estevez, J.M., Grant, M., and Molina, A. (2012). Disruption of abscisic acid signaling constitutively activates Arabidopsis resistance to the necrotrophic fungus *Plectosphaerella cucumerina*. *Plant Physiol.* 160**,** 2109-2124. doi: 10.1104/pp.112.200154

Suza, W.P., and Staswick, P.E. (2008). The role of JAR1 in Jasmonoyl-L-isoleucine production during *Arabidopsis* wound response. *Planta* 227**,** 1221-1232. doi: 10.1007/s00425-008-0694-4

Trabucco, G.M., Matos, D.A., Lee, S.J., Saathoff, A.J., Priest, H.D., Mockler, T.C., Sarath, G., and Hazen, S.P. (2013). Functional characterization of cinnamyl alcohol dehydrogenase and caffeic acid O-methyltransferase in *Brachypodium distachyon*. *BMC Biotechnol.* 13. doi: 10.1186/1472-6750-13-61

Wasternack, C., and Hause, B. (2013). Jasmonates: biosynthesis, perception, signal transduction and action in plant stress response, growth and development. An update to the 2007 review in Annals of Botany. *Ann. Bot.* 111**,** 1021-1058. doi: 10.1093/aob/mct067

Wurms, K., Gould, E., Ah Chee, A., Taylor, J., and Reglinski, T. (2013). Elicitor induction of defence genes and reduction of bacterial canker in kiwifruit. *Acta Phytopathol. Sin.* 43 (Suppl.)**,** 256-257.
